# Conserved Dynamic Mechanism of Allosteric Response to L-arg in Divergent Bacterial Arginine Repressors

**DOI:** 10.3390/molecules25092247

**Published:** 2020-05-10

**Authors:** Saurabh Kumar Pandey, Milan Melichercik, David Řeha, Rüdiger H. Ettrich, Jannette Carey

**Affiliations:** 1 Center for Nanobiology and Structural Biology, Institute of Microbiology, Czech Academy of Sciences, 37333 Nove Hrady, Czechia; pandey@nh.cas.cz (S.K.P.); mmelichercik@fmph.uniba.sk (M.M.); reha@nh.cas.cz (D.Ř.); 2 Department of Nuclear Physics and Biophysics, Faculty of Mathematics, Physics, and Informatics, Comenius University in Bratislava, 84248 Bratislava, Slovakia; 3 Faculty of Sciences, University of South Bohemia, 37005 Ceske Budejovice, Czechia; 4 College of Biomedical Sciences, Larkin University, Miami, FL 33169, USA; 5 Department of Cellular Biology & Pharmacology, Herbert Wertheim College of Medicine, Florida International University, Miami, FL 33199, USA; 6 Department of Chemistry, Princeton University, Princeton, NJ 08544, USA

**Keywords:** entropy, global motion, salt bridges, ligand binding, molecular evolution

## Abstract

Hexameric arginine repressor, ArgR, is the feedback regulator of bacterial L-arginine regulons, and sensor of L-arg that controls transcription of genes for its synthesis and catabolism. Although ArgR function, as well as its secondary, tertiary, and quaternary structures, is essentially the same in *E. coli* and *B. subtilis*, the two proteins differ significantly in sequence, including residues implicated in the response to L-arg. Molecular dynamics simulations are used here to evaluate the behavior of intact *B. subtilis* ArgR with and without L-arg, and are compared with prior MD results for a domain fragment of *E. coli* ArgR. Relative to its crystal structure, *B. subtilis* ArgR in absence of L-arg undergoes a large-scale rotational shift of its trimeric subassemblies that is very similar to that observed in the *E. coli* protein, but the residues driving rotation have distinct secondary and tertiary structural locations, and a key residue that drives rotation in *E. coli* is missing in *B. subtilis*. The similarity of trimer rotation despite different driving residues suggests that a rotational shift between trimers is integral to ArgR function. This conclusion is supported by phylogenetic analysis of distant ArgR homologs reported here that indicates at least three major groups characterized by distinct sequence motifs but predicted to undergo a common rotational transition. The dynamic consequences of L-arg binding for transcriptional activation of intact ArgR are evaluated here for the first time in two-microsecond simulations of *B. subtilis* ArgR. L-arg binding to intact *B. subtilis* ArgR causes a significant further shift in the angle of rotation between trimers that causes the N-terminal DNA-binding domains lose their interactions with the C-terminal domains, and is likely the first step toward adopting DNA-binding-competent conformations. The results aid interpretation of crystal structures of ArgR and ArgR-DNA complexes.

## 1. Introduction

Bacterial arginine repressor (ArgR) is the master regulator of the arginine regulon, sensing the intracellular concentration of L-arginine (L-arg) and exerting transcriptional control over synthesis of arginine biosynthetic and catabolic enzymes, including its own synthesis [1,2]. ArgR functions as a hexamer assembled as two trimers, with C-terminal domains (ArgRC) forming the core of the assembly and housing six L-arg binding sites, and peripheral N-terminal DNA-binding domains, each of which interacts with a C-terminal domain of another subunit across the trimer-trimer interface [3,4,5]. Although *E. coli* is the species with most available biochemical and genetic information in light of which structural data can be interpreted in terms of function, only separate N- and C-domain structures are available to date for *E. coli* ArgR, and a critical linker region implicated by genetic evidence is missing. Although the structures and functions of all characterized ArgRs are essentially identical, their sequence identities are unexpectedly low. For example, *B. subtilis* ArgR (BsArgR) can substitute for the function of *E. coli* ArgR (EcArgR) in vivo [6], but EcArgR and BsArgR share only ~27% sequence identity overall, with ~19% identity in the N-terminal domains, ~35% in the C-terminal domains, and distinct interdomain linker regions. Multiple alignment of EcArgR with sequences of available intact ArgR crystal structures produces results that misalign some of the secondary structures that define the highly conserved tertiary structures of the N- and C-terminal domains (Figure S1).

**Figure 1:**
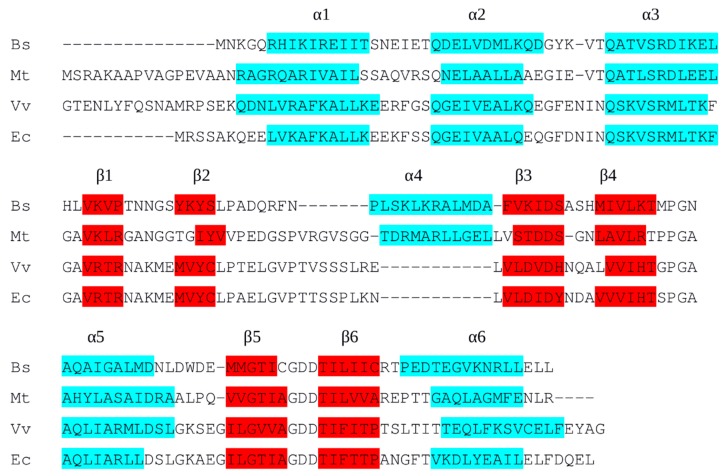
Structure-based sequence alignment. α-helices (cyan) and β-strands (red) are based on each respective PDB structure (BsArgR, PDB ID: 1F9N; MtArgR, PDB ID: 3FHZ; VvArgR, PDB ID: 3V4G; EcArgR: EcArgRN, PDB ID: 1AOY; EcArgRC, PDB ID: 1XXA). Although EcArgR and VvArgR structures may lack α4, numbering of their secondary structural elements counts the linker region as if α4 is present.

The definition and properties of the interdomain linker are unclear to date, yet they are directly relevant to understanding transcriptional activation by L-arg. Comparison of BsArgR and MtArgR crystal structures with and without bound DNA [5,7] implicates the interdomain region in considerable structural adaptation to accommodate the operator. Several mutants selected for altered EcArgR function in vivo map to the region between the folded N- and C-terminal domains [8], strongly suggesting that this region has an active role and is not merely a passive connector [9]. However, the low sequence conservation among ArgRs makes the boundaries between the domains and the linker difficult to assign with confidence (Figure 1), despite the availability of intact ArgR crystal structures from several organisms. The interdomain regions of *B. stearothermophilus* ArgR (BstArgR, Protein Data Bank (PDB) ID: 1B4A; [4]), *B. subtilis* ArgR (BsArgR, PDB ID: 1F9N; [5,10]), and *Mycobacterium tuberculosis* ArgR (MtArgR, PDB ID: 3FHZ, 3LAJ; [11,12]) present an irregularly structured segment followed by a 3-turn alpha helix, α4. In *E. coli*, however, ten of the 17 residues in the interdomain segment have low or very low helix propensity (Pro, Gly, Val, Thr, Ser, Asn), making a 3-turn alpha helix improbable. In crystals of intact ArgR from *Vibrio vulnificus* (VvArgR, PDB ID: 3V4G) with a linker-region sequence similar to that of EcArgR, many linker residues are missing due to presumed disorder, and the final three residues form one turn of 3_10_ helix.

Provisionally, the N- and C-terminal domains can be defined by the residues comprising the compact, well-ordered tertiary folds that are conserved in all known structures, with all intervening sequences considered to be part of the interdomain linker regardless of secondary structure. By this definition, the N-terminal DNA-binding domain ends with α2, and the C-terminal hexamerization and L-arg-binding domain begins with α3. The α4 helix of intact BstArgR has been assigned previously to the C-terminal domain based on inspection of the crystal structure [4]. The results described in the present work suggest for the first time a functional basis for inclusion of α4 as part of the C-terminal domain of those ArgRs in which it is present, and show how its function is maintained in ArgRs where it appears to be absent.

Crystal structures of the EcArgR C-terminal domain (EcArgRC) in the presence and absence of L-arg (apoEcArgRC with no L-arg bound and holoEcArgRC with all six L-arg bound) are essentially identical [3], showing only a very minor local difference distant from the ligand-binding site and thus offering no clue to the mechanism of response to co-effector binding. The hexameric structure observed in both apo- and holoEcArgRC domain crystals can be described as two trimers shaped as isosceles triangles stacked directly upon each other, with superimposed vertices corresponding to the centers of mass of each domain. Molecular dynamics simulations of apoEcArgRC [13] showed an immediate rotational shift of one trimer relative to the other compared with the directly stacked crystal structure, followed by rotational oscillation driven by coordinated formation and breakage of six hydrogen-bonded salt bridges. Arg110 in helix 5 from each subunit reaches across each empty L-arg binding site to contact Asp128 in the β-turn between strands 5 and 6 of the subunit that was superimposed on it in the initial unrotated structure (Table 1). MD simulations of holoEcArgRC [13] show that both the rotational shift and the rotational oscillations are significantly damped by the bound L-arg ligand, which displaces Arg110 and blocks salt-bridge formation. Surprisingly, in apoEcArgRC crystals the Cα atoms of the rotation-driving residues Arg110 and Asp128 are close enough to permit hydrogen bonding by the sidechain functional groups, yet these do not interact, but are surrounded by amorphous electron density [3] that likely represents counterions derived from the high salt content of the crystallization solution.

Crystal structures of intact *B. stearothermophilus* [4] and *B. subtilis* [5,10] apoArgRs also present an Asp residue in the turn between beta strands 5 and 6, but neither protein presents an Arg residue in helix 5, nor in any other location that could plausibly form a salt-bridge pair with it. Unlike *E. coli* ArgRC, both these *Bacillus* apoArgR structures differ from their holo crystal structures by a static rotation between trimers of approximately 15 degrees. *M. tuberculosis* apo and holo ArgRC crystals also differ by rotation of approximately 11 degrees [7]. MtArgRC presents residue Arg133 in helix 5, but compared to *E. coli* Arg110, Arg133 is one helical turn further away from Asp146, the residue corresponding to EcArgRC Asp128, and in neither rotated state would fully extended Arg133 reach fully extended Asp146 (data not shown). The origin of the static rotation observed in crystals of the *Bacillus* and *Mycobacterium* proteins is thus unclear. It could reflect the existence of dynamic rotational oscillation as seen in the EcArgRC simulations, or a functional requirement for static rotation, or an influence of crystal packing, perhaps together with the solution composition requirements for crystallization. Although crystal structures represent allowed states of molecules, they need not represent ground states, nor even highly populated states, as crystallization is not an equilibrium process.

These facts motivated the MD simulations reported in this work, which aimed to examine: the origins of the rotational difference observed between crystals of intact apo and holo BsArgR; whether this static difference reflects rotational oscillation as in EcArgRC, and, if so, to identify the residues driving it; and to try to understand the mechanism of transcriptional activation by L-arg by examining its influence on the N-terminal DNA-binding domains and interdomain linkers that are missing from EcArgRC, the only crystal structure available for the *E. coli* protein. BsArgR was chosen because the full-length protein is fully resolved in crystals. Although intact crystal structures are also available for VvArgR and MtArgR, VvArgR lacks critical residues in the linker region that are unresolved due to presumed disorder, and all intact MtArgR structures contain DNA, which imposes an initial state that might bias MD results on the free protein.

## 2. Results

### 2.1. Sequence Alignment

To gain insight into the definition, structure, and function of the region linking the N- and C-terminal domains of ArgRs, three ArgRs representing divergent interdomain sequences, *E. coli, B. subtilis,* and *M. tuberculosis,* were chosen for sequence analysis. The failure of simple sequence alignment to correctly align known secondary structural elements of the highly conserved ArgR domain structures (Figure S1) led to an attempt to adjust the alignment manually, respecting the conserved secondary structures common to all known ArgR domain structures as judged by their overlay in three-dimensional space in structural alignments of available ArgR crystal structures. Minimal gaps were introduced between secondary structural elements as needed to preserve structure alignment and maximize sequence conservation. The resulting alignment (Figure 1) is also fully consistent with the tertiary structures of the domains; i.e., the tertiary structure does not require any shift between the primary and secondary structures in the alignment. This finding further reinforces that the domain folds are very highly conserved despite the low overall sequence conservation.

The alignment reveals quite limited sequence conservation even within the well-aligned secondary structural elements, and it highlights significant differences in the region of the interdomain linker. BsArgR and MtArgR present irregularly structured segments of 8 and 14 divergent residues, respectively, followed by an alpha helix of 11 residues, α4. No more than one identical residue at a time can be aligned between the structurally aligned α4 helices conserved between the two organisms, in any frame of alignment. This fact suggests that α4 may have evolved independently in the two organisms, pointing to the possibility of evolutionary pressure to preserve a helix in this position. The positioning of EcArgR and VvArgR linker sequences with respect to α4 in Figure 1 is arbitrary, and no frame of alignment would more strongly support the existence of an α4 helix equivalent in these two. For simplicity, the secondary structures in all these ArgRs are numbered as if α4 is present. Because no structure is known for intact EcArgR it is formally possible that despite very low helical propensity, its linker region adopts a helical segment similar to BsArgR and MtArgR α4. However, it seems more likely that two main groups of ArgR linker types exist, with and without helix α4. If there is covariance elsewhere in the primary structure when α4 is present vs. absent, it is hardly evident from inspection of Figure 1. However, it may be significant that only EcArgR and VvArgR, which appear to lack α4, both have an Arg residue in α5. Arg110 in EcArgR is a key driver of trimer rotation, and BsArgR and MtArgR have no positional equivalent of EcArgR Arg110. Finally, the only completely conserved sequence of more than two residues that is common to all four ArgRs is GlyAspAsp in the tight turn between strands 5 and 6, containing Asp128 of EcArgR that interacts with Arg110 to drive rotation. The alignment of Figure 1 makes it even more remarkable that BsArgR can substitute for EcArgR function in vivo [6].

### 2.2. Phylogenetic Analysis

These results prompted further analysis of homologies among more distantly related bacterial ArgRs. The three ArgRs representing divergent linker regions, Ec, Bs, and Mt, were each used in a separate BLAST (**B**asic **L**ocal **A**lignment **S**earch **T**ool) search to identify its homologs independently of the other two ArgRs; VvArgR was not used to avoid bias due to its high similarity to EcArgR. The query sequence in each case was the full-length ArgR, i.e., each search was conducted without definition of any motifs or domains, nor with any specific focus on the linker region itself. Each search allowed for 20,000 hits, and among these hits each search also found the other two ArgRs with e-values in the range of 10^−20^ to 10^−30^, as expected from their limited sequence identities. From each search a set of homologs was then selected comprising the most distant sequences as judged by e-value that were still annotated as ArgRs, and excluding trivial repetitions such as isoforms or mutants, resulting in 29 distant homologs for EcArgR, 21 for BsArgR, and 34 for MtArgR. The final list comprises these 84 ArgRs. When taking EcArgR as the query the list spans an e-value range from 10^−100^ to 10^−7^ and a sequence identity range from 92% to 25%, thus representing a wide range of diversity of ArgRs. This set of 84 homologs was used to construct a phylogenetic tree using standard methods.

**Figure 2:**
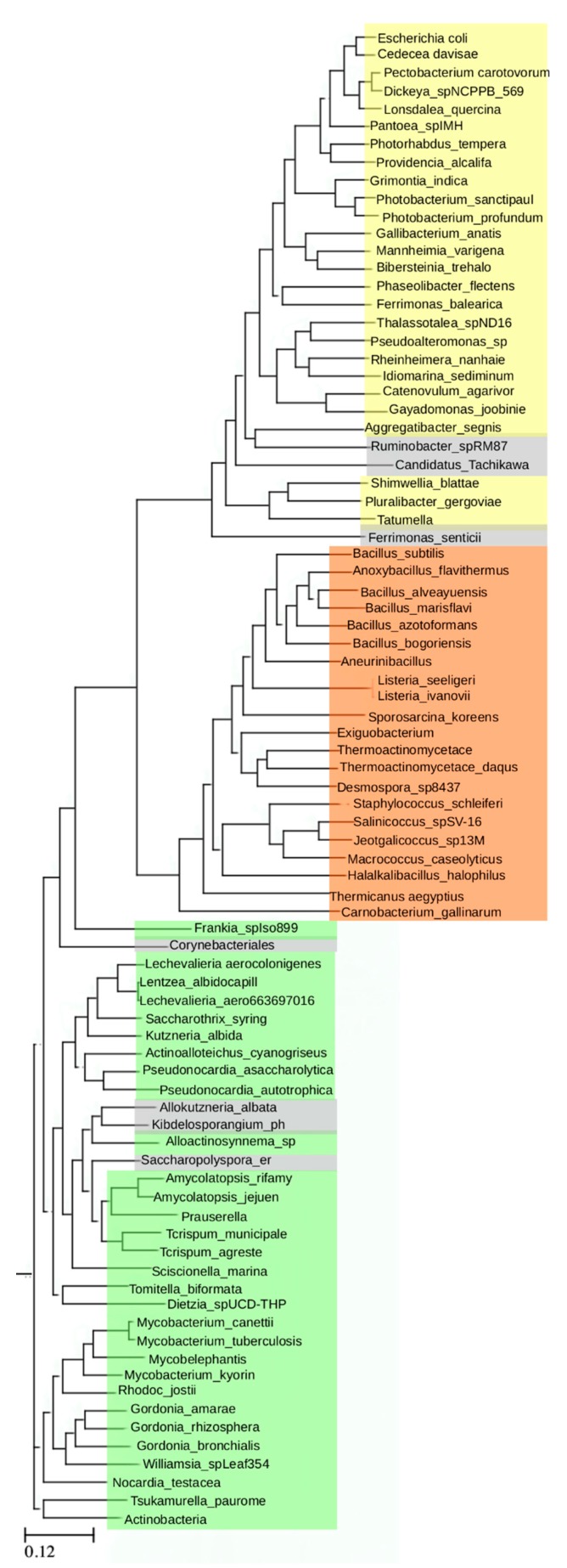
Phylogenetic tree of selected ArgR sequences. The three major branches are colored: green is defined by MtArgR and is closest in evolutionary distance to the common ancestor (scale bar, bottom, average number of residue substitutions per position); orange is defined by BsArgR; and yellow is defined by EcArgR. Entries shown in grey represent sequences that belong to the assigned phylogenetic group but lack the corresponding sequence motif.

The resulting tree (Figure 2) reveals three non-overlapping groups; these are defined by distinct sequence motifs. The EcArgR group is defined by a motif in the L-arg binding site, _107_LIA**R**--D_113_ in α5, that includes Arg110 (bold), one of the two residues (with Asp128) driving trimer rotation in apoEcArgRC. This motif is shared by VvArgR, as shown in Figure 1. The BsArgR and MtArgR groups share related but distinct sequence motifs in α4 of the linker region, _75_K--R---D_82_ in BsArgR and _86_R--R---E_103_ in MtArgR. The pattern of spacing of these charged residues is conserved, and the motifs align in the structure-based sequence alignment of Figure 1. The three motif groups are mutually exclusive, i.e., linker regions in the EcArgR group have no sequences corresponding to the α4 motif, and α5 helices in the BsArgR and MtArgR groups do not have the EcArgR-group sequence motif, although the equivalent of EcArgR Asp128, but not Arg110, is present in most. A few outliers (grey in Figure 2) in the BsArgR or EcArgR groups lack the motif according to simple sequence analysis, suggesting that more than three groups may exist, as does the fact that even an Asp128 equivalent is missing in some. The finding that the BsArgR and MtArgR groups lack an equivalent of Arg110 suggests that the trimer rotation observed in the crystal structures of their canonical members, BsArgR and MtArgR, has a different origin than the rotation observed by MD in apoEcArgRC.

### 2.3. Molecular Dynamics Simulations

**Figure 3:**
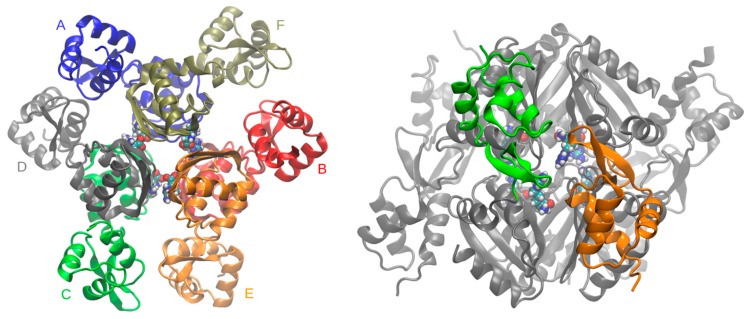
Model of holoBsArgR. The model was prepared as described in the text by docking six molecules of L-arg in the empty binding sites of the apoBsArgR crystal structure (PDB ID: 1F9N). Monomers are shown in cartoon representation in unique colors and labeled A–F. Atoms of L-arg are shown as spheres with atomic colors and cyan carbons. (**Left**) Top view down the 3_2_ axis. The central C-terminal domains form two trimers stacked directly atop one another to form a hexameric core, and pairs of peripheral N-terminal DNA-binding domains (CE, green-orange; BF, red-tan; and AD, blue-grey) can be identified. (**Right**) Side view, rotated from the left panel by 90° about the z-axis and enlarged. This view shows that L-arg ligands are located at the trimer-trimer interface, and N-terminal domain pairs form across the trimer interface, illustrated for the green and orange CE subunits; all remaining subunits are in grey for clarity.

In the earlier molecular dynamics simulations of the *E. coli* protein that revealed its rotational oscillation, only the oligomerization domains (EcArgRC) could be analyzed, as no full-length intact structure of EcArgR was available then (nor even now; PDB, February, 2020). When starting this comparative work on *B. subtilis* it seemed therefore logical to analyze its oligomerization domains only. However, when apoBsArgRC oligomerization domains (prepared from the intact apoBsArgR crystal structure (PDB ID: 1F9N) by removing the N-terminal domains with or without the linker region) were simulated, the hexamers soon become unstable (data not shown); thus no equilibrium state could be reached with apoBsArgRC, and all simulations were instead performed using the intact apoBsArgR structure (PDB ID: 1F9N), thus offering for the first time the possibility to also evaluate the dynamic mechanism of transcriptional activation by L-arg. To represent holoBsArgR a structural model was prepared in silico by superimposing the crystal structure of apoBsArgR (PDB ID: 1F9N) on that of holoBsArgRC (PDB ID: 2P5M) to guide placement of six molecules of L-arg manually in the six empty binding sites at the trimer-trimer interface (Figure 3), followed by energy minimization. Docking was facilitated by the fact that the BsArgR L-arg binding site contains no resident Arg residue equivalent to EcArgR Arg110, which in apo EcArgRC blocks access to the ligand. The local structures of apoBsArgR and holoBsArgRC are essentially identical, enabling placement of L-arg ligands into the empty binding sites of apoBsArgR by superposition with holoBsArgRC. Consistent with this fact, both apo- and holoBsArgR were well-behaved in simulations of up to 2 µsec.

**Figure 4:**
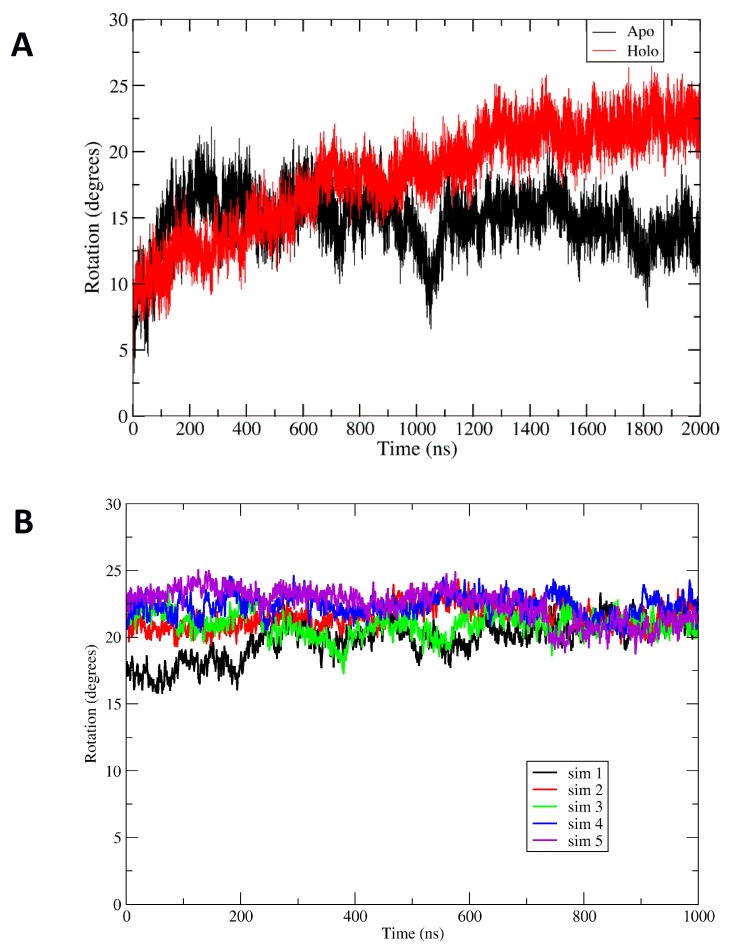
Global rotational shift of BsArgR. (**A**) Each initial structure (0 time) undergoes a very fast initial rotational shift of ~10 degrees, then evolves by further rotation, reaching a mean value of rotation during the last 500 ns of the equilibrated part of the trajectories of 14 ± 1.4 (mean ± std. deviation) degrees for apoBsArgR and 22 ± 1.3 degrees for holoBsArgR. Rotation angle is calculated as depicted in Figure S6. As described in the text, the variance observed in each time course is uncoordinated motions of each subunit, not rotational oscillation of trimers. (**B**) Sim 1 represents the equilibrated holoArgR simulation from 1–2 us from panel A but with averaging over a 1-ns window. Sims 2–5 are replicas of that simulation starting from the equilibrated holoBsAgR conformation, each averaged over a 1-ns window.

Comparison of the apoBsArgR and holoBsArgR trajectories shows that the two systems differ from each other and from the starting structures. For both proteins, a rotational shift of one trimer with respect to the other occurs very early, followed by continued evolution of the rotational angle toward an equilibrium value that persists for up to 3 µsec (Figure 4). Trimer-trimer rotation was quantified using an in-house script [14] as described in Methods. The initial degree of rotation in apoBsArgR crystals is 1.2° (and no crystal structure is available for holoBsArgR, which therefore has the same initial rotation as apoBsArgR from which it was prepared). During 2 µsec simulations both apo- and holoBsArgR show a clockwise rotational shift about the 3_2_ axis compared to the initial structure. In the last 500 ns of the trajectories the average degree of rotation is ~14° for apoBsArgR and ~22° for holoBsArgR. The difference of ~8 degrees between apo- and holoBsArgR is similar to the difference between apo- and holoEcArgRC reported in MD simulations [14].

However, unlike EcArgRC, where the apoprotein exhibits strong rotational oscillation that is driven by formation and release of Arg110–Asp128 salt bridges across the empty ligand-binding site and is damped upon binding of L-arg, both apo- and holoBsArgR display no rotational oscillation. This finding is consistent with the fact that BsArgR has no residue corresponding to EcArgRC Arg110. The variance of ± 5–6 degrees observed in the time courses of Figure 4 is not rotational oscillation of trimers relative to each other. Principal components analysis and calculation of the eigenvectors show that the motions contributing to the observed variances correspond to uncoordinated motions of individual subunits and not to rotation. This result comports with PCA analysis of *E. coli* ArgR systems (Strawn et al., 2010). The uncoordinated motions reflect wobbling motions of monomers within trimers, a more complex and rapid multidimensional movement that contributes to apparent variation in the calculated angle of rotation; this interpretation is supported by results shown below for a mutant that does not undergo rotation. The more extreme deviations from mean rotational angle observed for apoBsArgR reflect slow motions that permit the very large system to occasionally sample states with limited rotation (~1020 ns) and states with holo-like rotational angles (~1500 ns).

**Figure 5:**
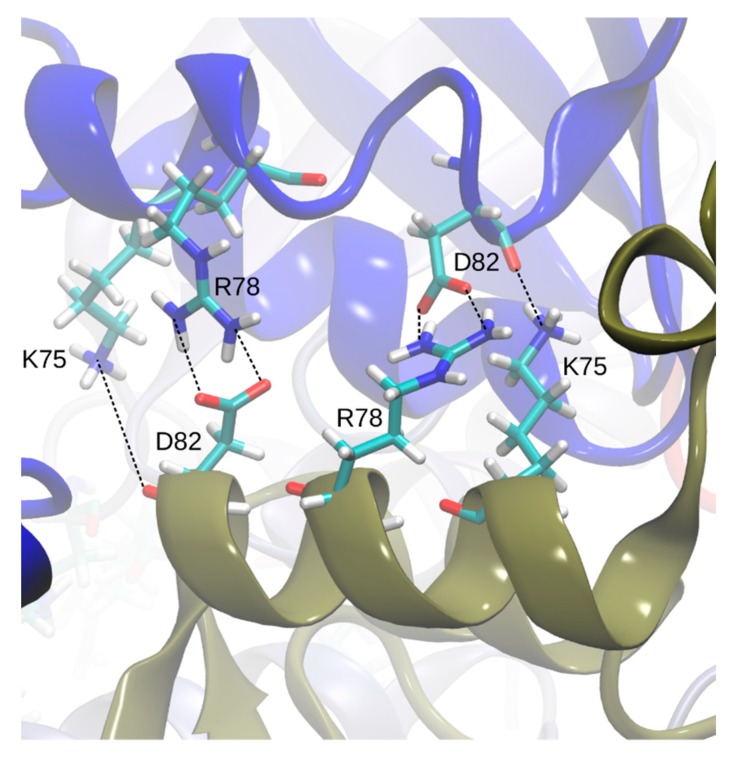
Inter-trimer interactions. Residues Lys75, Arg78, and Asp82 (in one-letter code) on helix α4 (blue), and their symmetry-equivalent residues on antiparallel helix α4′ from the closest subunit on the other trimer (tan), are shown as stick models in atomic colors with cyan carbons. Dashed lines show all hydrogen-bonded salt bridges that form during the simulation, although not all are within hydrogen-bonding distance simultaneously (as shown in Figure 6); e.g., in this snapshot taken from 1903 ns, the distance between the left-most Lys75–Asp82 pair is longer than hydrogen-bonding distance.

Visual screening of the apo- and holoBsArgR trajectories for potential rotation-driving interactions suggested that the linker-region sequence motif that defines the Bs phylogenetic branch (K75--R78---D82, Table 1) plays a role in the rotational shift. Over the course of both apo- and holoBsArgR trajectories, inter-trimer interactions form between these residues located on helix α4 from a subunit on one trimer and the symmetry-equivalent residues of antiparallel helix α4′ from the closest subunit in the other trimer (Figure 5). The interactions comprise salt bridges between oppositely charged residues that are brought within hydrogen-bonding distance by rotation: Lys75: Nζ with Asp82: Oδ; Lys75: Nζ with Asp82: O; and Arg78 Nη with Asp82: Oδ. In the initial state before any rotation occurs (i.e., at zero time in Figure 4), the sidechain functional groups of these residues are too far apart to be consistent with hydrogen-bond formation, as observed in the apoBsArgR crystal structure from which both protein systems were prepared, even though the distance between their corresponding Cα atoms would permit hydrogen bonding by the extended functional groups of these long sidechains. The unexpected observation that fully charged sidechains within hydrogen-bonding distance do not interact echoes that in apoEcArgRC, where Arg110 and Asp128 sidechains are close enough to form a doubly hydrogen-bonded salt bridge across each empty L-arg binding site, but do not do so [3], which has been suggested to reflect the very high salt concentration during crystallization [13]. In holoBsArgRC (PDB ID: 2P5M) the sidechains of Arg78 and Asp82 are ~3 Å apart and thus can be presumed to interact, although this crystal structure was refined as a trimer, and the hexamer was prepared by a symmetry operation [10].

**Figure 6:**
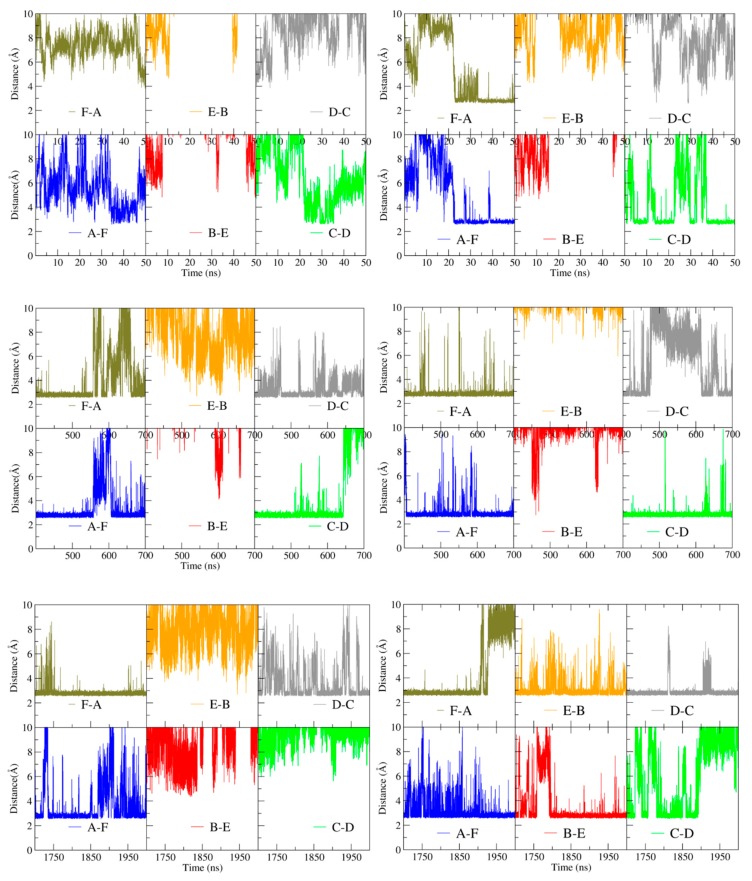
Interactions driving rotation. Left, apoBsArgR; right, holoBsArgR. Colors correspond to subunits in Figure 3. Distance between guanidino nitrogen atoms of Arg78 and carboxylate oxygen atoms of Asp82 is plotted for subunit pairs during three time windows of the rotational time course shown in Figure 4; these distances over the full time course are presented in Figure S2. Letters in lower right of each panel indicate the subunit pairs whose inter-residue distances are measured as identified in Figure 3. The first 50 ns of the simulations is shown in the top panels; 400–700 ns is shown in the middle panels; the final 300 ns is shown in the bottom panels.

The identity of the interacting residues at the inter-trimer interface does not differ between apo- and holoBsArgR, consistent with the fact that helix α4 is far removed from the L-arg binding site (~17 Å throughout the simulations). However, the total number and persistence of inter-trimer interactions between the α4 residues increase over the course of both simulations, correlated with the degree of rotation. The interactions were quantified by monitoring the distance between the guanidino nitrogen atoms of Arg78 and the carboxylate oxygen atoms of Asp82 (Figure 6) over the time course of Figure 4A. Figure 6 presents three selected time ranges that represent different extents of rotation; the full time course is shown in Figure S2. The minimum possible inter-atom distance, ~3 Å, corresponds to hydrogen-bond occupancy.

In the first ~20 ns of the simulations, when only the initial rotational shift of ~10 degrees has occurred, very few close-approach distances occur for either protein, and none is persistent (upper panels). Between ~20 and ~50 ns, as rotation begins to evolve, some close-approach distances are populated, especially in holoBsArgR. Between 400 and 700 ns (middle panels) as rotation continues to evolve with a common mean rotational angle for both proteins in Figure 4A, Arg78–Asp82 hydrogen-bond occupancies are also similar in both proteins, with four of the six possible bonding pairs formed most of the time. During the final 300 ns of each trajectory (lower panels), when each protein achieves its full extent of rotation in Figure 4A, hydrogen-bond occupancies reach their maximum for holoBsArgR with ~22 degrees rotation and nearly full bond occupancy (seven interactions in total [data not shown], four Arg78–Asp82 sidechain and three Lys75–Asp82 backbone), and with ~14 degrees rotation and partial occupancy for apoBsArgR (three Arg78–Asp82 sidechain interactions). The much lower number of trimer-trimer interactions in apoBsArgR may explain the hexamer instability that was observed in early simulations lacking the N-terminal domains.

In addition to residues of the α4 helix, the L-arg ligand plays a direct role in enlarging the rotational angle of holoBsArgR to its fullest extent. Before the rotational angle reaches ~15 degrees the distance between the Cα carbon of Asp125 (the equivalent of EcArgR Asp128) and the position of the L-arg guanidino group is too large for interaction with the Asp125 sidechain, which faces toward the solvent (data not shown). As the rotational angle approaches ~20 degrees the Asp125 sidechain begins to point toward the position of the L-arg guanidino group in holoBsArgR simulations only. At this point the electrostatic complementarity and rotation angle begin to act in parallel in holoBsArgR, resulting ultimately in a doubly hydrogen-bonded salt bridge between the Asp125 carboxylate and the L-arg guanidino group, and with a maximum rotational angle of ~22 degrees. These results indicate that the trimer-trimer interface residues in the α4 helix, and their mutual interactions promoting rotation, are linked to L-arg binding, as expected from the fact that the ligand-binding sites span the trimer interface. Importantly, however, to reach the maximum degree of rotation and full salt-bridge occupancy, the N-terminal domains must be liberated from their interactions with the C-terminal domains, as discussed below.

If the interactions between residues of the α4 helix indeed drive the rotation observed in apo- and holoBsArgR, then eliminating the interacting residues is expected to eliminate trimer-trimer rotation. To test this prediction the triple-mutant Lys75Ala/Arg78Ala/Asp82Ala, in which all three charged sidechains are replaced by the alanine methyl group, was prepared in silico and simulations were performed for mutant apo- and holoBsArgR. Both apo and holo triple mutants show a much smaller initial rotational shift (~5 degrees) with little or no further evolution of the mean rotational angle over ~1 µs (Figure S3). The small initial shift may reflect minor optimization of trimer-trimer interactions relative to the crystal structure. The variance observed in Figure 4 for wildtype BsArgR is also observed for the triple mutant, affirming that it is not due to rotational oscillation, which occurs in neither mutant nor wildtype BsArgR. In the holoBsArgR triple mutant, two L-arg ligands leave the binding site within the first 100 ns of simulation, and one more ligand leaves after 200 ns (data not shown), indicating that the system is not in an L-arg binding-competent state and suggesting that in further time all six L-arg ligands may dissociate. This result can be understood by considering that when wildtype holoBsArgR is prepared by docking L-arg into the apoBsArgR structure, the ligand finds a binding-competent conformation among the conformational ensemble, and the sampled conformational space can further approach binding-compatible conformations via the rotational shift. When the rotational shift does not occur, as in the triple mutant, L-arg binding-compatible conformations remain rare.

### 2.4. Consequences of L-arg Binding

**Figure 7:**
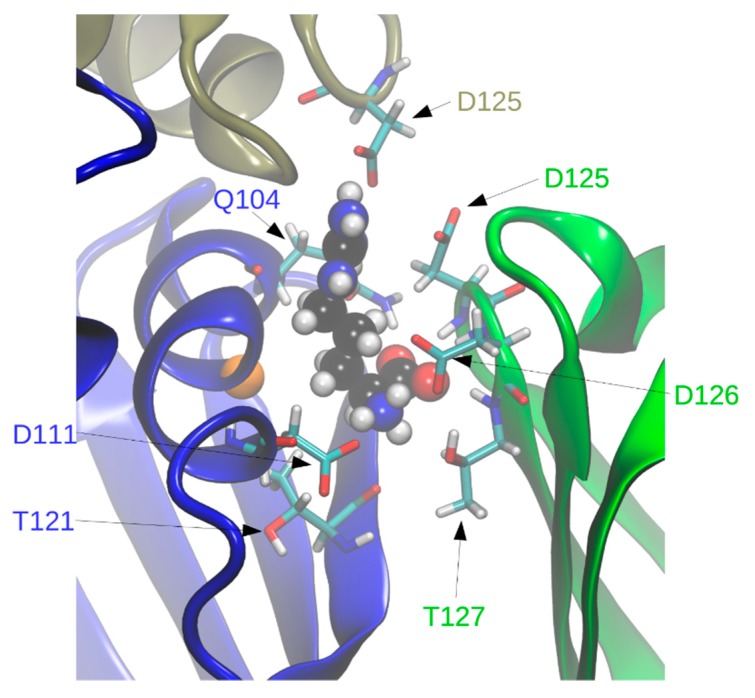
L-arginine binding-site interactions. The three subunits that form representative binding site A, shown, are colored according to Figure 3. Site A is defined as the binding site adjacent to helix α5 of chain A (blue) and is formed by chains A and C (green) from one trimer and F (tan) from the other trimer. Residues whose sidechain functional groups are within hydrogen-bonding distance of the ligand are shown as sticks with atomic colors and black carbons. Residue numbers (one-letter code) are colored according to their subunit of origin. L-arg is shown in CPK spheres with atomic colors and cyan carbons. The position of the Gly107 alpha carbon is indicated by an orange sphere.

In holoBsArgR simulations each L-arginine ligand is surrounded by residues from three subunits that form each binding site (Figure 7), consistent with the crystal structure. Seven of the surrounding residues present numerous sidechain functional groups that are appropriate for hydrogen bonding with functional groups of the L-arg ligand, and ten of these functional groups remain within hydrogen-bonding distance during much of the simulation time (Table 2). The fact that L-arg forms multi-dentate contacts with multiple subunits is similar to the case of the *E. coli* protein, although the identity of contacting residues is different except for Asp125 and Asp126, the aligned sequence equivalents of EcArgR Asp128 and Asp129 (compare Figure 1). Unlike the *E. coli* case, no resident Arg residue equivalent to EcArgR Arg110 is displaced by ligand binding to BsArgR. In holoEcArgR the very extensive network of interactions surrounding the alpha-amino and alpha-carboxylate groups of the L-arg ligand enables the free amino acid to compete effectively with resident residue Arg110 despite the far greater local effective concentration of a covalently attached residue. For both Bs and EcArgR, multi-subunit contacts provide a direct structural means for signaling among subunits upon L-arg binding.

The fact that each ligand has extensive contacts with three subunits spanning both trimers suggests that the binding of L-arg contributes to the stability of the hexameric assembly. Indeed, not only L-arg but also the N-terminal domains and α4 helix appear to be involved in hexamer stabilization, as suggested by the following results with BsArgRC. HoloBsArgRC trimers stay associated, and hexamers remain symmetric, during the entire simulation periods, whether or not the α4 helix is present (data not shown). In contrast, the apoBsArgRC hexamer including α4 does not equilibrate, and although trimers do not separate, the hexamer becomes increasingly asymmetric due to the wobbling motions described above. In apoBsArgRC without the α4 helix, hexamers become asymmetric even more rapidly, and trimers separate within the first 100 ns. These findings may be related to previously published reports that some ArgRs crystallize, and even bind to DNA in solution, as trimers [4,7,15,16,17,18].

Interestingly, in BsArgR the position structurally equivalent to *E. coli* Arg110 in helix α5 is occupied by Gly107 (Figure 7). This observation is consistent with the speculation advanced previously for ArgR [13], and supported experimentally for the *E. coli* tryptophan repressor [19,20], that binding sites for amino acid ligands can develop when mutations replace protein residues corresponding to the free amino acid. Such a step in the early evolution of ArgR as the master feedback regulator of the arginine regulon might thus be captured in the comparison of Bs, Ec, and Mt ArgRs presented here. However, the fact that all three ArgRs respond functionally to L-arg binding, even though EcArgR retains residue Arg110 and the others do not, makes it difficult to evaluate which organism may be more similar to the evolutionary progenitor, and which is more similar to the progeny.

### 2.5. Interactions between N- and C-Terminal Domains

Visual analysis of trajectories reveals that by the end of the simulations the N-terminal domains occupy positions that differ between apo- and holoBsArgR and from each starting structure, and with little or no internal rearrangement within either N-terminal or C-terminal domains. To quantify the domain interactions, distances were measured between potential hydrogen-bonding pairs that can span the N- and C-terminal domains, with distances less than 3 Å during the final 500 ns of the simulations and populated more than 50% of the time scored as interdomain hydrogen-bonding interactions. The identities of the residues and their atoms that come within hydrogen-bonding distance are listed in Table 3. Of the eight hydrogen-bond pairs that are sampled in apoBsArgR, only three are also sampled in holoBsArgR, and in fewer subunits in all cases. One hydrogen-bond pair, between Arg78 and His49, is sampled uniquely in holoBsArgR, but in only one subunit per hexamer. None of the interactions comprise hydrogen-bonded salt-bridge pairs; rather, each involves at least one uncharged polar backbone atom. Thus, the orientation of N- and C-terminal domains observed in apoBsArgR is enforced by the collective effect of many weak interactions rather than by a few dominant ones. Consequently, once the angle of rotation grows large enough upon L-arg binding, all the weak N- to C-terminal domain bonds are compromised together, releasing the N-terminal domains. It is notable that none of the residues involved in interactions between the N- and C-terminal domains of BsArgR is conserved in EcArgR, which is also consistent with the predominance of backbone atoms in the interactions. Even between BsArgR and Mt ArgR, which share a similar pattern of charged residues in helix α4, some of which are involved in interdomain interactions, no homologous residues are found among the hydrogen-bonding pairs in Table 3 (data not shown).

**Figure 8:**
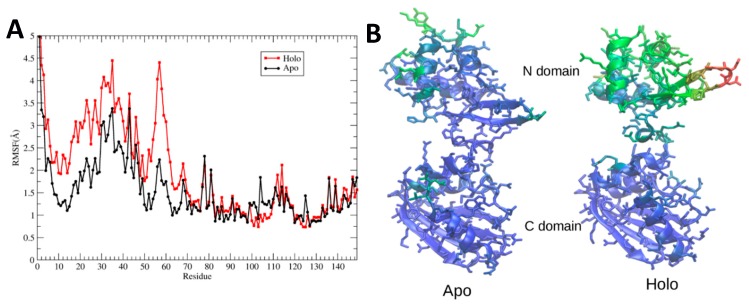
Domain movements upon L-arg binding. (**A**) RMSF was calculated for all non-hydrogen atoms during the final 500 ns of each simulation and averaged over the six monomers of the hexamer. (**B**) RMSF calculated for representative monomer F was converted to crystallographic B-factors (see Methods) and colored from lowest (blue) to highest (red).

These results are consistent with visual inspection of the trajectories, which indicates that when L-arg is bound the N-terminal domains are more mobile and have moved away from the C-terminal domains. Figure 8A quantifies this behavior by plotting the maximum root-mean-square fluctuations of heavy atoms of apo- and holoBsArgR during the final 500 ns of each simulation. The results clearly show large fluctuations N-terminal to linker residue 66, and very limited fluctuations beyond this point. Root mean square fluctuation (RMSF) values are larger overall for the N-terminal domains of holoBsArgR by approximately 1 Å, and largely parallel to those of apoBsArgR except in the turn between strands 1 and 2, where the RMSF difference is approximately 2.5 Å. The fact that RMSFs for apo- and holoproteins change in parallel supports the view that the N-terminal domains move as units, because parallel RMSFs are not expected if internal structural change occurs. Figure 8B displays on apo- and holoBsArgR structures the B-factors calculated from the corresponding RMSF values. This comparison shows that in addition to the overall increase in their mobility, the N-terminal domains of holoBsArgR shift slightly away from the C-terminal domains. These results, together with the finding that the triple mutant does not rotate, strongly suggest that rotation is indeed the cause of the smaller number of interactions in holoBsArgR that permits greater mobility of its N-terminal domains compared to apoBsArgR.

This interpretation is also supported by analysis of root mean square deviation (RMSD) values calculated for the whole hexamer and for the individual domains (Figure S4). Figure S4A shows that although RMSD values for the C-terminal domain are in the range 1–1.5 Å typically expected in an equilibrated protein system, global domain movements cause RMSD values of ~3 Å for apoBsArgR, and values above 5 Å for holoBsArgR. Thus, RMSD does not reflect fluctuations of internal secondary or tertiary structure within the domains. However the result does suggest that the N-terminal domains of apoBsArgR either are much less mobile than those of holoBsArgR, or that if they are not more mobile then they must have other changes to account for the RMSD. These two possibilities are resolved in Figure S4B, where superposition of individual domains removes translational and rotational domain movements. Increased RMSD values in the N-terminal domains are instead correlated with fluctuations in the turn between helices α2 and α3, as can be seen by comparing with the RMSF values in Figure 8. Repositioning of these helices accounts for the RMSD value of ~3Å. Because there is clearly no global unfolding or other changes to the internal domain structure during the simulations, the RMSD results confirm that in holoBsArgR the N-terminal domains move as units, and more freely than in apoBsArgR.

Liberation of the N-terminal domains increases the entropy contribution to the free energy of the system, further contributing to the stability of holoBsArgR. To quantify the entropic differences, the contribution of configurational entropy to the total free energy of each system was calculated from the trajectories as described in Methods. The configurational entropy contribution is increased for holoBsArgR (81,094 ± 3087 J/mol K) compared to apoBsArgR (72,693 J/mol K). This result presumably reflects the higher flexibility observed for the N-terminal domains of holoBsArgR even though lower entropy contributions are expected from the C-terminal domains due to the extensive bonding between each L-arg ligand and three of the six the subunits. Indeed, in holoBsArgR the contribution of the N-terminal domains is higher than in apoBsArgR, 46378 ± 1718 J/mol K vs. 40,445 J/mol K. However, the entropy contribution of the C-terminal domains of holoBsArgR is also increased relative to apoBsArgR, 38,787 ± 1705 J/mol K vs. 35521 J/mol K, although the difference between apo- and holoBsArgR C-terminal domain contributions is smaller than for their N-terminal domains (a gain of 3266 J/mol K for the C-terminal domains vs. 5933 J/mol K for the N-terminal domains). Thus, the increase in entropy of holoBsArgR compared to apoBsArgR is not evenly distributed throughout the protein; rather, the N-terminal domains contribute disproportionately, ~60% of the total entropy gain, although the C-terminal domains also contribute ~40% of the total entropy gain. Please note that the method for calculating entropy contributions of domains and intact proteins does not necessarily result in additive values.

### 2.6. Approaching DNA-Binding-Competent Conformations

The conformational differences observed between holoBsArgR and apoBsArgR in the positions of their DNA-binding domains may be related to the mechanism of activation of DNA by L-arg binding. To evaluate this possibility the structure of holoBsArgR in the final 500 ns of the simulation was compared with known requirements for DNA binding based on biochemical evidence for EcArgR [9,21,22] and crystal structures of other ArgR-DNA complexes [7,10,11,12]. In a crystal structure of two isolated N-terminal domains of BsArgR bound to a short palindromic DNA duplex (PDB ID: 2P5L) the major groove in successive openings on one “face” of the DNA is occupied by two N-terminal domains, with key residue Arg43 of each domain contacting symmetry-equivalent guanine residues of the palindromic sequence. In this structure the distance between C-alpha carbons of the two Arg43 residues is ~26 Å, consistent with footprinting and stoichiometric binding data indicating that one ArgR N-terminal domain binds to each half-palindrome, and that pairs of domains bind to each full palindrome [9,21,22]. Those biochemical data also indicate that the tandem palindromes of typical operators in the arg regulon engage four of the six subunits of an ArgR hexamer, requiring positioning of two pairs of N-terminal domains to allow contact with four successive openings of the DNA major groove on one face. The distance between C-alpha carbons of Arg43 residues in N-terminal domain pairs of ArgR was therefore measured over the course of the simulations for apoBsArgR and holoBsArgR.

**Figure 9:**
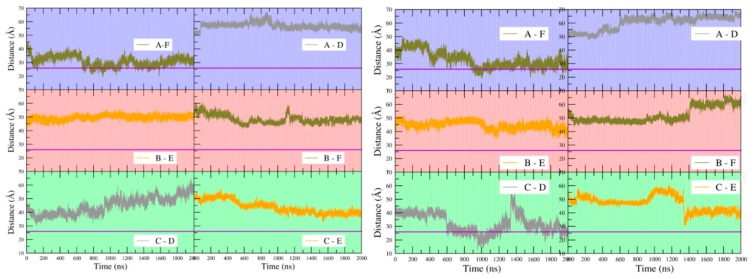
Distance between N-terminal domain pairs. The distance between Cα atoms of residue Arg43 in neighboring N-terminal domains was calculated during the time course of the simulations. The background color of each panel corresponds to one monomer of one trimer (A, B, or C; blue, red, or green) as in Figure 3. The colored lines on each panel indicate the N-terminal domain from the other trimer (D, E, or F; grey, orange, or tan) as in Figure 3. Left six panels, apoBsArgR; right six panels, holoBsArgR.

Figure 9 shows that at the beginning of the simulations the distance between Arg43 C-alpha carbons on any pair of subunits is in the range of ~40 to ~50 Å for both apo- and holoBsArgR, consistent with the crystal structure of apoBsArgR from which holoBsArgR was prepared, where the initial distance is ~40 Å. This result shows that the multiple bonding interactions between the N- and C-terminal domains of apoBsArgR (Table 3) position the domains too far apart for DNA binding. The interdomain distances become smaller or larger over time, and with no obvious covariance between domain pairs for either apo- or holoBsArgR. In apoBsArgR the distance approaches ~26 Å occasionally only for monomers A and F (Figure 9 left). This finding suggests that apoBsArgR may be on its way toward a DNA-binding-competent state that would be constitutively active for binding to a single palindrome. In holoBsArgR the distance between Arg43 C-alpha carbons on any pair of subunits approaches ~26 Å for subunit pairs A–F and C–D (Figure 9 right), indicating structures that may be on the way toward binding-competent states for the tandem palindromes of natural Arg operators. In neither apo- nor holoBsArgR is the position of the N-terminal domains fully optimal for DNA binding, however, as helix α3 must also be oriented approximately parallel to the base pair planes in the DNA major groove opening according to known crystal structures. Whole domain movements are expected to be slow (on a μs–ms rather than ns–μs timescale), and on a longer timescale the conformations sampled by holoBsArgR might progress toward DNA-binding-competent states. Whether or not they do so, proximity to a DNA segment bearing suitably spaced palindromic sequences is expected to enforce the distances and orientations required for binding.

## 3. Discussion

The results presented here reveal that arginine repressors from *E. coli* and *B. subtilis* share a common global motion, a relative rotation of the two trimers comprising the hexamer, even though rotation is driven by different residues in distinct locations in the two proteins. In the *E. coli* ArgRC domain the rotational motion is not static but is accompanied by large-scale rotational oscillations back and forth. The present results on intact BsArgR suggest that the N-terminal domains limit its motion to a static change in rotation angle, suggesting that the oscillations observed in EcArgRC could reflect the absence of its N-terminal domains; this possibility cannot be evaluated until a crystal structure of intact EcArgR is available as a starting point for MD simulations. However, binding of the co-effector ligand L-arg has quite distinct consequences in the two proteins, blocking the oscillatory motion in EcArgRC when the first ligand binds, but promoting an increase of the rotational angle in BsArgR. The possibility that intact EcArgR also responds to L-arg binding with an increase of rotational angle as in BsArgR, instead of with blocked oscillation as in EcArgRC, appears to be ruled out by the location and known interactions of L-arg in holoEcArgRC. The striking similarity of the motions despite their very different drivers and consequences suggests that trimer-trimer rotation is integral to ArgR function.

The present results thus indicate that the static rotation observed in BsArgR and BsArgRC crystal structures in fact reflects a critical functional property, and not a rare state trapped by requirements of crystal growth. Extrapolation of these conclusions further suggests that the static rotation observed in crystal structures of *B. stearothermophilus* ArgR [4] and *M. tuberculosis* ArgR and ArgRC [7,11,12] is likely to reflect a similar functional rotation, driven by residues in the interdomain helix that are homologous to those of BsArgR, and with analogous predicted consequences upon L-arg binding. A final implication is that the crystal structures of apo- and holoEcArgRC, in which no rotation between trimers is observed and the salt-bridging groups that drive rotation are close enough to bond but do not, likely represent a rare state trapped during crystal growth. This inference serves as a reminder that although crystal structures represent states that can occur, they may offer little or no information about critical functional dynamics.

Given the profound differences between EcArgR and BsArgR in the drivers and consequences of their rotation, the common factor underlying a functional requirement for rotation may be that it promotes motion of the N-terminal domains upon L-arg binding, as found here for BsArgR and inferred previously for EcArgR based on results for EcArgRC. The disposition of the N-terminal DNA-binding domains in the crystal structures of BsArgR is incompatible with DNA binding, and mobility allows the domains to explore orientations that may lead to DNA-binding-competent states. Although for the *E. coli* protein no intact ArgR structure is available with which to evaluate this proposal by further MD simulations, the earlier results of EcArgRC simulations are consistent with it. Simulations of EcArgRC in the presence and absence of one L-arg ligand per hexamer [13] showed an increase in B-factors at the surface of the C-terminal domain upon binding of the first L-arg ligand. Increased B-factors were traced to frustration of rotational oscillation caused by contradictory effects: the bound ligand spans the trimer interface at one binding site, which blocks oscillation, but in the remaining five empty sites the Arg110–Asp128 charge pairs are still attracted across the empty binding sites, which would promote oscillation. This frustration imparts a shuddering motion to the domain hexamer that is expressed as increased mobility of residues at its surface because the center of the assembly is relatively immobile due to the extensive network of bonding interactions made by the single ligand to three subunits. The increased mobility at the EcArgRC hexamer domain surface is likely to be propagated to the N-terminal domains, resulting in their increased mobility as observed in holoBsArgR, but by a fundamentally different mechanism.

The results of the present work support reported structures of some ArgR-DNA complexes in which the DNA-bound N-terminal domains attach to DNA oligomers rather than remaining associated with the C-terminal domains [7,10,11,12]. However, for EcArgR such structures appear to be ruled out by available biochemical results showing that each single palindromic DNA is bent by ~35 degrees upon protein binding, which is inferred to require cooperation between two N-terminal domains [22] that may be incompatible with flexibly attached DNA-binding domains. The presence, length, and sequence of DNA can alter, and even direct, the crystallization of DNA-binding proteins, as exemplified by many cases including the tryptophan repressor, which co-crystallizes with short DNAs as either a single dimer [23] or a cooperative pair of dimers [24], depending on the local sequence context of its binding site. Like some tryptophan repressor crystals, many protein-DNA cocrystals are highly anisotropic, with coaxial alignment of DNA oligomers facilitated by base stacking across the duplex termini. This tendency suggests that crystal-packing requirements, perhaps together with the typically unusual solution conditions for crystal growth, may conspire to influence the structure of protein-DNA complexes in ways that are not evident. These considerations amplify the fact that crystal structures represent allowed states of systems, but not necessarily ground states or other highly populated states.

L-arg binding to both EcArgR and EcArgRC is strongly negatively cooperative, with the first ligand binding ~100-fold more strongly than the remaining five, as shown by isothermal titration calorimetry [25]. MD showed that binding of the first L-arg ligand to apoEcArgRC takes advantage of frequent opening of one ligand-binding site in the oscillating hexamer [14]. Once one ligand is bound and rotation is frustrated, the frequency of opening a second binding site is reduced, consistent with the lower affinity after the first binding event. When taken together with the present results indicating that liberation of N-terminal domains enables them to explore DNA-competent conformations, a functional interpretation can now be suggested for the negatively cooperative L-arg binding to EcArgR and EcArgRC. The increased mobility at the surface of all six C-terminal domains induced by binding of the first L-arg ligand to one subunit is likely to be sufficient to liberate all six N-terminal domains, suggesting that the singly liganded state of EcArgR may be activated for DNA binding. It is also possible that the DNA-binding activity of singly ligated EcArgR differs from that of fully ligated ArgR; either or both the affinity and the specificity for its DNA targets [26] may differ. Regrettably, experiments aimed at evaluating DNA binding by singly liganded ArgR have been unsuccessful to date despite exhaustive attempts by numerous methods (J.C., unpublished observations). Thus, the functional relevance of the pronounced negative cooperativity determined experimentally for EcArgR binding to L-arg remains to be established with certainty, even if its structural and dynamic basis has been revealed by MD simulations.

Although exhaustive effort has also been given to crystallize intact EcArgR (J.C., unpublished), the failure to discover crystals, taken together with the facile proteolytic liberation of its N-terminal domains [21] and crystallization of EcArgRC by unintended proteolysis of EcArgR [3], may signal that its N-terminal domains are less well-localized on the C-terminal domains than in BsArgR. Less well-localized N-terminal domains of EcArgR might also be expected from the poor structure-forming propensity predicted for its interdomain sequence. Such mobile domains may explore DNA-binding-competent states independently of L-arg, which might be reflected in relatively strong apoprotein DNA-binding affinities. Indeed, the affinity (dissociation equilibrium constant, K_d_) of apoEcArgR for operator DNA is relatively strong, ~300 nM, only ~60-fold weaker than the affinity (K_d_, 5 nM) of holoEcArgR [22]; and the affinity for non-operator DNA is even less dependent on binding of L-arg, with K_d_ values of 10 and 1 uM, respectively. These values for affinity of holoEcArgR reflect occupancy by all six L-arg ligands; as noted above, it has not yet been possible to determine the affinity of singly ligated EcArgR for operator or non-operator DNA.

No information is available from experiment or simulation to suggest how the N-terminal domains of BsArgR respond to partial occupancy of L-arg ligands in the C-terminal domains. The effects of single-ligand binding to BsArgR have not yet been examined by MD and are beyond the scope of the present work, but the success of docking six L-arg ligands here suggests that removal of five ligands from the equilibrated holoBsArgR structure is a suitable starting point for a future investigation. L-arg binding has apparently not yet been studied experimentally for ArgRs other than EcArgR, which may be related to the difficulty of quantifying L-arg binding to ArgR. ArgRs generally lack chromophores or fluorophores in or near the ligand-binding sites, and L-arg binding to EcArgR cannot be detected by any spectroscopic means (UV-Vis, CD, fluorescence; J.C., unpublished observations). This result is consistent with the MD finding that ligand binding alters EcArgRC rotational oscillation without any significant change in internal structure of domains or subunits. The altered rotation observed in MD for BsArgR upon L-arg binding, which also occurs without accompanying internal structural changes, is also not expected to lead to any observable change in spectroscopic signals. Calorimetry may be a useful way to quantify L-arg binding to BsArgR.

Although it is unknown whether BsArgR binds L-arg with negative cooperativity, the ecological niche of *B. subtilis* differs from that of *E. coli* in ways that are likely to offer distinct selective pressure to respond to intermediate L-arg levels. *E. coli* experiences regular cycles between feast-and-famine levels of L-arg in the human gut that likely invokes switching between biosynthesis and catabolism of L-arg, both of which are regulated by EcArgR. In contrast, *B. subtilis* is a soil organism whose environmental exposure to L-arg is almost certainly much less volatile. These significant biological differences may be reflected in differential quantitative responses to L-arg in the Bs- and EcArgR systems.

## 4. Materials and Methods

The phylogenetic tree of 84 sequences was built using clustalw2_phylogeny with neighbor-joining for clustering and visualized in ETE [27]. Sequences were chosen via BLAST search using the *E. coli*, *B. subtilis,* or *M. tuberculosis* sequence as query using the BLOSOM62 matrix with gap costs 11 (existence), 1 (extension), a word size of 6, and an expect threshold of 1.0. From the results, distant relatives were chosen to exclude biasing the results by weighting with more similar sequences. The multiple sequence alignment was calculated in Omega [28]. In addition to the multiple sequence alignment calculated by Omega using standard parameters for the four sequences with available crystal structures (*E. coli*, *B. subtilis*, *V. vulnificius* and *M. tuberculosis*), a structural alignment using the Mustang [29] implementation in Yasara [30] was calculated to guide manual adjustment of the misaligned linker region and the first helix of the C-domain.

ApoBsArgRC crystal structure (PDB entry 1F9N) was cleaned from crystallization additives in Yasara [30] and solvated in TIP3P water [31]. Because there is no crystal structure of intact holoBsArgR or any closely related species, the holoBsArgRC domain (PDB ID: 2P5M) was superimposed on the intact apoBsArgR structure (PDB ID: 1F9N) to minimize distances between corresponding atoms, and ligands were added to the intact apo structure. As the parameters for free L-arg ligand are not available in the Amber99SB force field, parameterization for L-arg was carried out using the standard **R**estrained **E**lectro**s**tatic **P**otential (RESP) procedure [32], with charges derived from HF/6-31G* calculation [33] of free L-arg in zwitterionic form. The charges were nearly identical (data not shown) to those calculated previously for L-arg with capped alpha substituents [34]. In silico point mutations were carried out in Yasara to prepare the triple-mutant Lys75Ala/Arg78Ala/Asp82Ala.

Molecular dynamics analysis used the modeling package GROMACS 5.1 [35,36]. The simulation cell extended 2.0 nm beyond the protein and periodic boundary conditions were applied. The system was neutralized with potassium and chloride ions at concentration 0.1 mol/L. For simulations, the Amber99SB-ILDN force field [37,38,39] was employed. Electrostatics were evaluated using the particle-mesh Ewald method [40] with a cutoff of 1.0 nm; van der Waals forces were evaluated with a Lennard–Jones potential with a 1.0 nm cutoff. Velocity rescale thermostat [41] and Berendsen barostat [42] were employed (coupling constants 0.1 ps), with the protein and solvent atoms in separate baths maintained at 300 K, and pressure maintained at 1 bar with compressibility 4.6 × 10^−5^/bar. Time steps of 2 fs were used. The solvated system was first energy minimized using steepest descent and the solvent allowed to relax. Initial Boltzmann-weighted velocities were generated randomly, and the system was further equilibrated for 500 ps while keeping the protein restrained. The MD production runs without constraints were carried out up to 2 μs for wildtype protein and to at least 1 μs for the triple mutant, which appeared to stabilize after shorter simulation time. For apoArgR a second independent simulation was initiated and simulated for 500 ns, which replicated the fast rotational shift in the beginning and the attainment of equilibrium after 200 ns (Figure S5). A holoArgR structure, the result of the 2 μs holoArgR simulation, was used to initiate 4 repetitions from conformations observed in the equilibrated phase, and simulated for 1 μs each.

Analyses of MD results were done using standard GROMACS utilities. For calculating the angle of rotation only the ligand-binding domain was used. The rotation of one trimer over the other trimer was analyzed by the same in-house script described previously for *E. coli* [14]. The center of mass of monomers was calculated over Cα atoms of the C-terminal domain using residues 85–149, and then rotation of centers of mass of the two trimers over each other was measured as depicted in Figure S6. The hexamer was shifted and rotated until centers of mass (CoM) of trimers were located at the z-axis. During the simulations, the trimers stay in parallel horizontal planes, so z-axis movements could be neglected. Then angles are calculated between vectors CoM(ABC)–CoM(A) and CoM(DEF)–CoM(F), CoM(ABC)–CoM(B) and CoM(DEF)–CoM(E), CoM(ABC)–CoM(C) and CoM(DEF)–CoM(D), respectively, where A–F are subunit identities as in the crystal structure and the nearest pairs of monomers are A–F, B–E, and C–D as in Figure 3. The final angle of rotation is the average of those three vectors. Graphs were produced in Grace [43] and figures in VMD [44].

Entropies were computed from covariance matrices produced by g_covar using a quasi-harmonic approximation [45] implemented in gmx anaeig in GROMACS. Differences of the conformational entropy were calculated by quasi-harmonic analysis from k_B_/2 ln(det σ_a_/det σ_b_), where det σ_a_ and det σ_b_ are covariance matrices of atomic fluctuations, and k_B_ is Boltzmann’s constant. To gain higher accuracy the original Schlitter’s approximation [46] was improved by removing the singularity of the covariance matrix in Cartesian coordinates [47]. Entropy calculations were carried out over the last 1 μs of the trajectories. Frames were sampled every 1 ps, well beyond the minimum frame number required for quasi-harmonic approximations [47], yielding results that are independent of frame number. Conversion of RMSF to B-factors used blue-green-red scaling in VMD [48] with offset 0.10 and midpoint 0.28. For these calculations, the N-terminal domain is defined as residues 1 to 71, representing the structured N-terminal domain plus the unstructured part of the interdomain linker. The C-terminal domain is defined as residues 72 to 149, representing the interdomain linker helix α4 and the entire C-terminal domain.

Global motions of domains were analyzed using principal components analysis. Covariance matrices of atomic positions in the trajectories were calculated and diagonalized using gmx covar in GROMACS. The trajectory was projected on eigenvectors of the covariance matrix using the gmx anaeig tool. The extreme positions along eigenvectors were extracted and visualized in VMD [44].

## Figures and Tables

**Table 1:** ArgR interdomain and rotation-driving sequences.

ArgR; PDB ID	Interdomain Sequence ^a^	Interdomain Secondary Structure	Rotation Holo/Apo Crystal	Rotation Holo/Apo MD	Residues Driving Rotation ^b,c^	Rotation-Driving Residues’ Location ^b,c^	References
Bs 1F9N 2P5M	_64_LPADQRFNPLS**K**LK**R**ALM**D**A_83_	α4	15°	9°	K75/R78/D82A and K75′/R78′/D82A’	α4 and α4′	[10] this work
Ec 1AOY 1XXA	_69_LPAELGVPTTSSPLKNL_85_	unknown	0°	5°	R110 and D128′	α5 and β5′-β6′ turn	[13,14]
Mt 3FHZ 3BUE	_80_VPEDGSPVRGVSGG TD**R**MA**R**LLG**E**LLV_106_	α4	11°	unknown	R97/R100/E104 and R97′/R100′/E104′	α4 and α4′	[7] this work

^a^ Interdomain residues (one-letter code) are defined as described in the text. Residue numbers are taken from each respective PDB file. Helical segments are indicated in cyan. Residues in bold comprise the motifs characteristic of the corresponding phylogenetic group identified in Figure 2. ^b^ Residue numbers and locations without a prime mark are in one subunit, and with a prime mark are in a second subunit. ^c^ MtArgR and BsArgR residues driving rotation are predictions based on the present work.

**Table 2:** Interactions with L-arginine.

	Monomer A (Blue) ^a^	Monomer C (Green)	Monomer F (Tan)
L-arg binding site A	Gln104 Nε: L-arg O Gln104 Oε: L-arg Nη Asp111 Oδ: L-arg N Thr121 O: L-arg N	Asp125 N: L-arg O Asp126 N: L-arg O Asp126 Oδ: L-arg N Thr127 Oγ: L-arg N	Asp125 Oδ: L-arg Nη

^a^ Colors correspond to Figure 3 and Figure 7.

**Table 3:** Interdomain hydrogen-bond interactions.

Interaction ^a^	Apo ^b^	Holo ^b^
11Arg(N)–81 Met(O)	4	
11Arg(Nη)–84 Phe(O)	3	1
66Ala(N)–82 Asp(O)	3	
68Gln(Nε)–100Pro(O)	1	
75Lys(Nζ)–65 Pro(O)	2	1
75Lys(Nζ)–67 Asp(O)	3	2
112Asn(Nδ)–16 Ser(O)	1	
102Asn(Nδ)–66 Ala(O)	2	
78Arg(Nη)–49 His(O)		1

^a^ A hydrogen-bond interaction is scored for each distance <3 Å with more than 50% occupancy during the final 500 ns of each trajectory. ^b^ The number of interactions observed per hexamer; each interaction has a maximum value of six, i.e., one bond of the indicated type per subunit. To simplify tabulation, not all listed pairs are necessarily within one subunit.

## Supplementary Materials

The following are available online. Figure S1: Multiple sequence alignment. This alignment was not adjusted and used Clustal Omega with standard parameters. Symbol definitions: *, identical residues; chemically interchangeable residues as defined by scoring >0.5 in the Gonnet PAM 250 matrix; chemically similar residues as defined by scoring ≤0.5 in the Gonnet PAM 250 matrix. Compare Figure 1. Figure S2: Interactions driving rotation. Left, apoBsArgR; right, holoBsArgR. Colors correspond to subunits in Figure 3. Distance between guanidino nitrogen atoms of R78 and carboxylate oxygen atoms of D82 is plotted for subunit pairs during the full time course shown in Figure 4. Letters in lower right of each panel indicate the subunit pairs whose inter-residue distances are measured as identified in Figure 3. Figure S3: Global motions in triple-mutant Lys75Ala/Arg78Ala/Asp82Ala. Following an initial rotational shift from each starting crystal structure both apo- and holoprotein systems do not evolve significantly, reaching a common mean value of rotation ~7 ± 1.5 degrees (mean ± std. dev.). Figure S4: RMSD evaluation of domain motions. RMSD values were calculated every 20 ps for the last 200 ns of the 2 us simulation shown in Figure 4. Subunit colors are the same as in Figure 3. (**A**) Hexamer superposition. The Cα atoms of each indicated intact hexamer were superimposed on the first frame in the calculation (i.e., at 1.8 μs), and RMSD was calculated separately for the N- and C-terminal domains. (**B**) Domain superposition. The Cα atoms of each indicated domain were superimposed on those from a representative frame from the equilibrated phase (1.5 μs), and RMSD was calculated for each domain. Upper panels (A and B), N-terminal domains; lower panels (C and D), C-terminal domains; left panels (A and C), holoBsArgR; right panels (B and D), apoBsArgR. Figure S5: Global rotational shift of apoBsArgR. A 500 ns independent replica of the apoBsArgR simulation (green) in comparison with the simulation reported in Figure 4 (black) to assess the reproducibility of the observed behavior. Figure S6: Calculation of rotation angle. The C-terminal domains of each ArgR subunit (colored circles) are located at the apexes of two triangles (solid and dashed lines). Colored circles represent centers of mass of these C-domains (residues 85–149) of the three monomers in each layer of the ArgR hexamer; the layers are shown as slightly offset for clarity but the two trimeric layers are directly stacked. The angle between two triangles in the apoBsArgRC crystal structure is initially zero as shown in the upper panel, as the two trimers stack directly upon each other. To calculate the rotation angle in each frame of the trajectory a triangle representing the centers of monomer mass in that frame was superimposed on the starting structure. Using g_traj and an in-house script (14), the angle of each monomer’s center of mass with respect to its corresponding initial position was calculated (blue shaded areas at center) and the average of the resulting deviation of the angles was assigned as the value of rotation.
