# Supplementary material for: Conserved Dynamic Mechanism of Allosteric Response to L-arg in Divergent Bacterial Arginine Repressors
Source: Molecules. 2020 May 10;25(9):2247. doi: 10.3390/molecules25092247 (PMC7248756; doi:10.3390/molecules25092247)
Supplement: Supplementary file 1 [file molecules-25-02247-s001.pdf]

Conserved Dynamic Mechanism of Allosteric Response to L-arg in Divergent Bacterial Arginine Repressors

Saurabh Kumar Pandey [1,2,3,†], Milan Melichercik [1,2,†], David Řeha [1,3], Rüdiger H. Ettrich [1,4,5,*] and Jannette Carey [1,6,*]

1. Center for Nanobiology and Structural Biology, Institute of Microbiology, Czech Academy of Sciences, 37333 Nove Hrady, Czechia; pandey@nh.cas.cz (S.K.P.); mmelichercik@fmph.uniba.sk (M.M.); reha@nh.cas.cz (D.Ř.)
2. Department of Nuclear Physics and Biophysics, Faculty of Mathematics, Physics, and Informatics, Comenius University in Bratislava, 84248 Bratislava, Slovakia
3. Faculty of Sciences, University of South Bohemia, 37005 Ceske Budejovice, Czechia
4. College of Biomedical Sciences, Larkin University, Miami, FL 33169, USA
5. Department of Cellular Biology & Pharmacology, Herbert Wertheim College of Medicine, Florida International University, Miami, FL 33199, USA
6. Department of Chemistry, Princeton University, Princeton, NJ 08544, USA

* Correspondence: jcarey@princeton.edu (J.C.); rettrich@ularkin.org (R.H.E.); Tel.: +1-609-258-1631 (J.C.); +1-954-682-8347 (R.H.E.)

† These authors contributed equally to this work.

Supplemental Figures:

```
Bs              ---------------MNKG----QRHIKIREIITSNEIETQDELVDMLKQDGYK-VTQAT    40
Mt              MSRAKAAPVAGPEVAANRA----GRQARIVAILSSAQVRSQNELAALLAAEGIE-VTQAT    55
Vv              ----GTENLYFQSNAMRPSEKQDNLVRAFKALLKEERFGSQGEIVEALKQEGFENINQSK    56
Ec              ---------------MRSSAKQEELVKAFKALLKEEKFSSQGEIVAALQEQGFDNINQSK    45
                                . .         :  ::.. .. :*.*:.  *  :* . :.*:.

Bs              VSRDIKELHLVKVPTNNGSY-KYSLPADQR-----FNPLSKLKRALMDAFVKIDSASHMI    94
Mt              LSRDLEELGAVKLRGADGGTGIYVVPEDGSPVRGVSGGTDRMARLLGELLVSTDDSGNLA    115
Vv              VSRMLTKFGAVRTRNAKMEM-VYCLPTELG--------VPTVSSSLRELVLDVDHNQALV    107
Ec              VSRMLTKFGAVRTRNAKMEM-VYCLPAELG--------VPTTMSPLKNLVLDIDYNDAVV    96
                :** : ::  *:    .     * :* :                 * : .:. *    :

Bs              VLKTMPGNAQAIGALMDNLDW-DEMMGTICGDDTILIICRTPEDTEGVKNRLLELL----    149
Mt              VLRTPPGAAHYLASAIDRAAL-PQVVGTIAGDDTILVVAREPTTGAQLAGMFENLR----    170
Vv              VIHTGPGAAQLIARMLDSLGKSEGILGVVAGDDTIFITPTLTITTEQLFKSVCELFEYAG    167
Ec              VIHTSPGAAQLIARLLDSLGKAEGILGTIAGDDTIFTTPANGFTVKDLYEAILELFDQEL    156
                *::* ** *: :.  :*       ::*.:.*****:           :   . :*
```

Figure S1: Multiple sequence alignment using Clustal Omega with standard parameters. Symbol definitions: *, identical residues; :, chemically interchangeable residues as defined by scoring > 0.5 in the Gonnet PAM 250 matrix; ., chemically similar residues as defined by scoring ≤ 0.5 in the Gonnet PAM 250 matrix. Compare Figure 1.

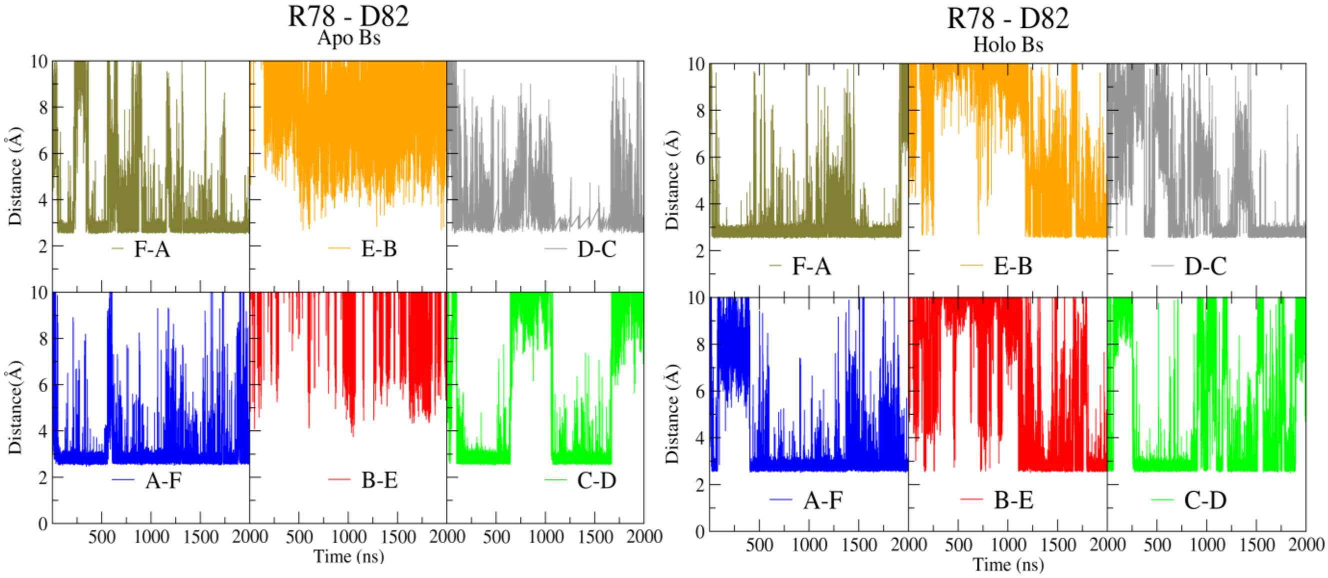

Figure S2. Interactions driving rotation. Left, apoBsArgR; right, holoBsArgR. Colors correspond to subunits in Figure 3. Distance between guanidino nitrogen atoms of R78 and carboxylate oxygen atoms of D82 is plotted for subunit pairs during the full time course shown in Figure 4. Letters in lower right of each panel indicate the subunit pairs whose inter-residue distances are measured as identified in Figure 3.

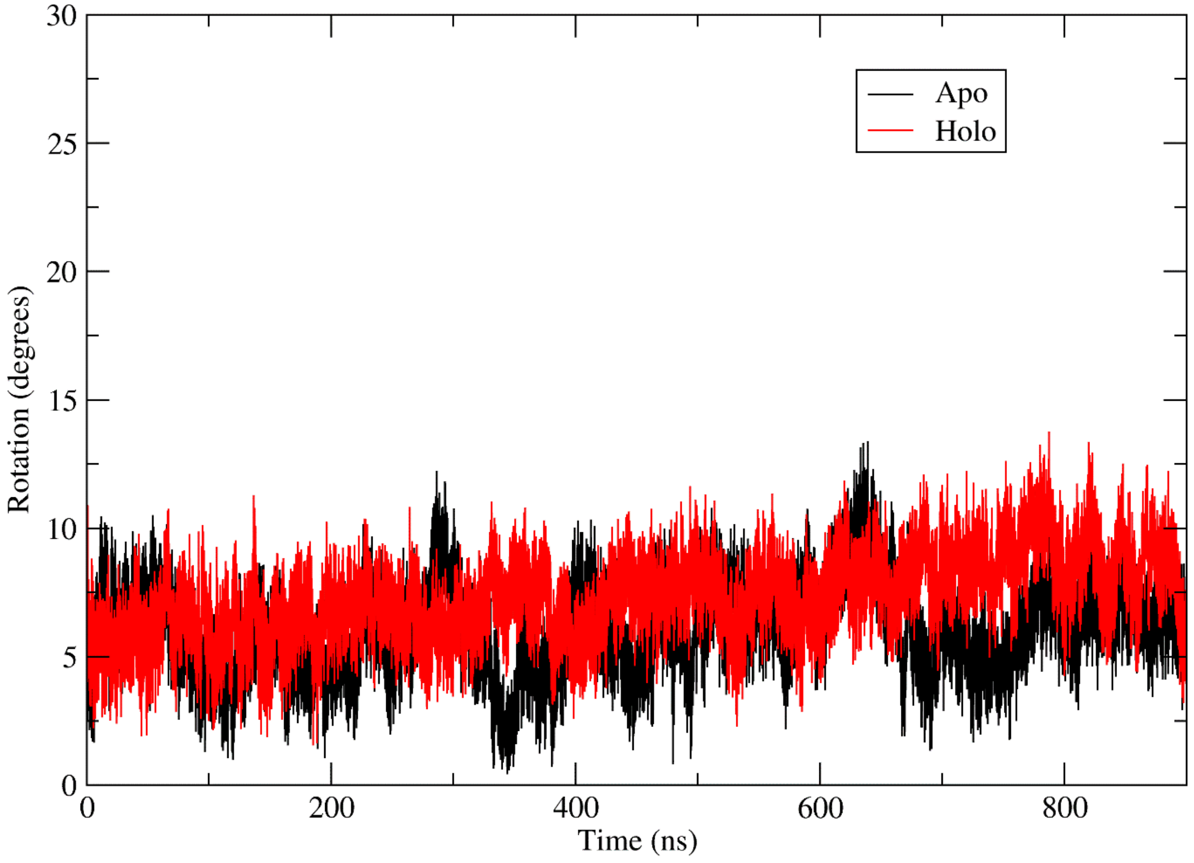

Figure S3. Global motions in triple-mutant Lys75Ala/Arg78Ala/Asp82Ala. Following an initial rotational shift from each starting crystal structure both apo- and holoprotein systems do not evolve significantly, reaching a common mean value of rotation ~7 ± 1.5 degrees (mean ± std. dev.)

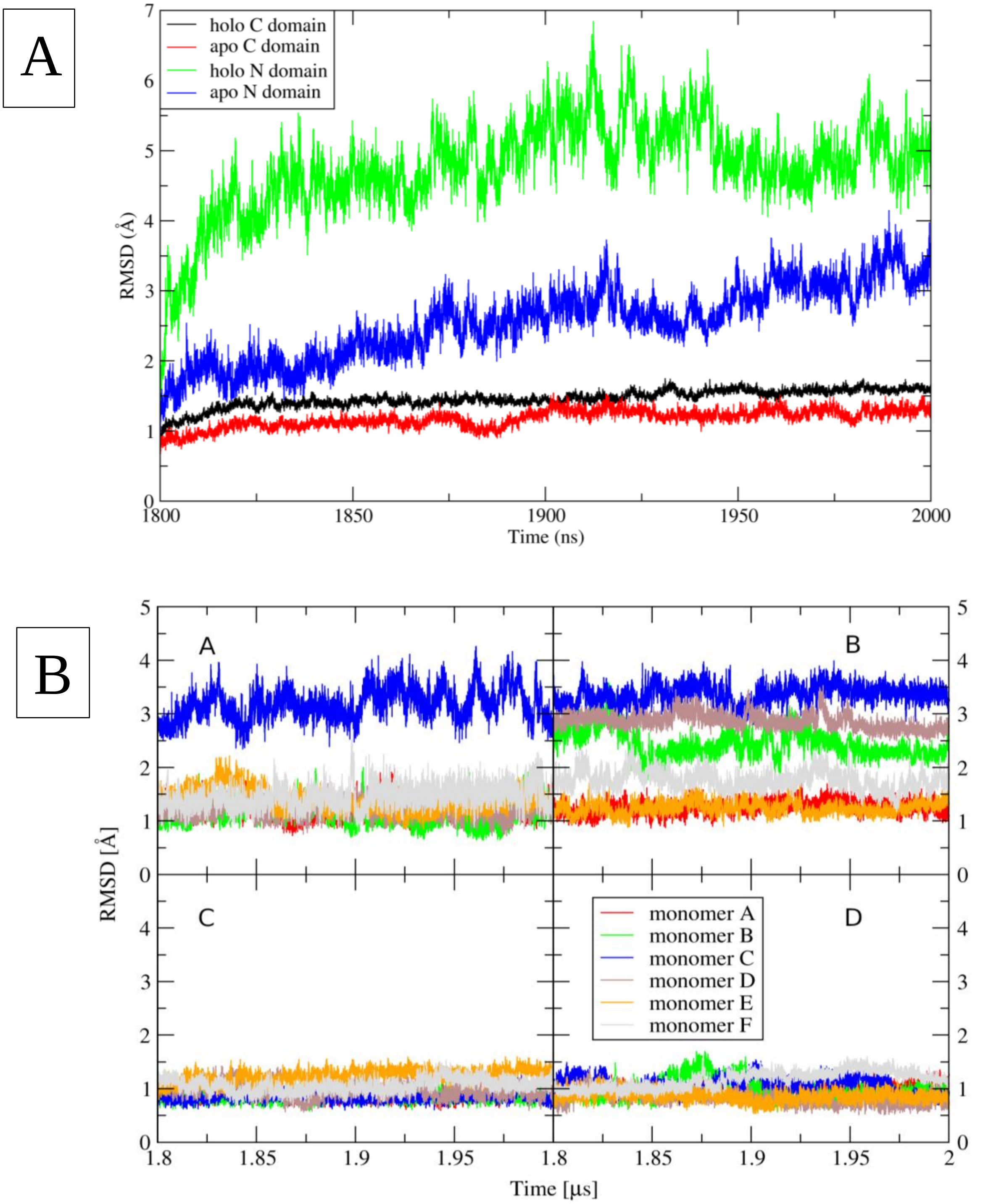

Figure S4. RMSD evaluation of domain motions. RMSD values were calculated every 20 ps for the last 200 ns of the 2 us simulation shown in Figure 4. Subunit colors are the same as in Figure 3. **A. Hexamer superposition.** The Cα atoms of each indicated intact hexamer were superimposed on the first frame in the calculation (i.e., at 1.8 μs), and RMSD was calculated separately for the N- and C-terminal domains. **B. Domain superposition.** The Cα atoms of each indicated domain were superimposed on those from a representative frame from the equilibrated phase (1.5 μs), and RMSD was calculated for each domain. Upper panels (A and B), N-terminal domains; lower panels (C and D), C-terminal domains; left panels (A and C), holoBsArgR; right panels (B and D), apoBsArgR.

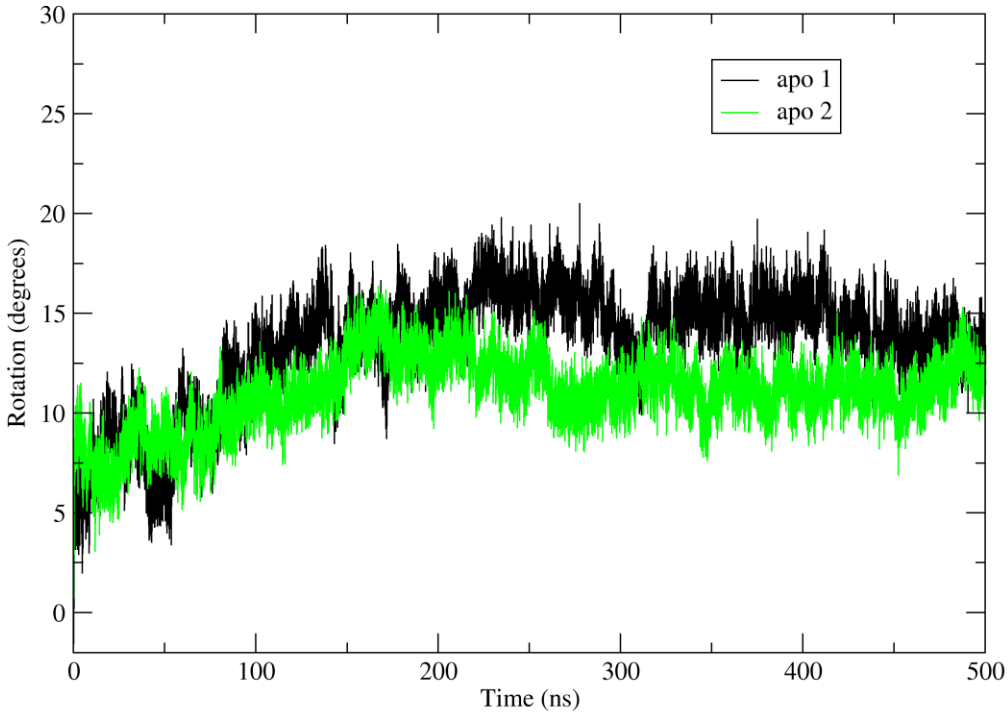

Figure S5. Global rotational shift of apoBsArgR. A 500 ns independent replica of the apoBsArgR simulation (green) in comparison with the simulation reported in Figure 4 (black) to assess the reproducibility of the observed behavior.

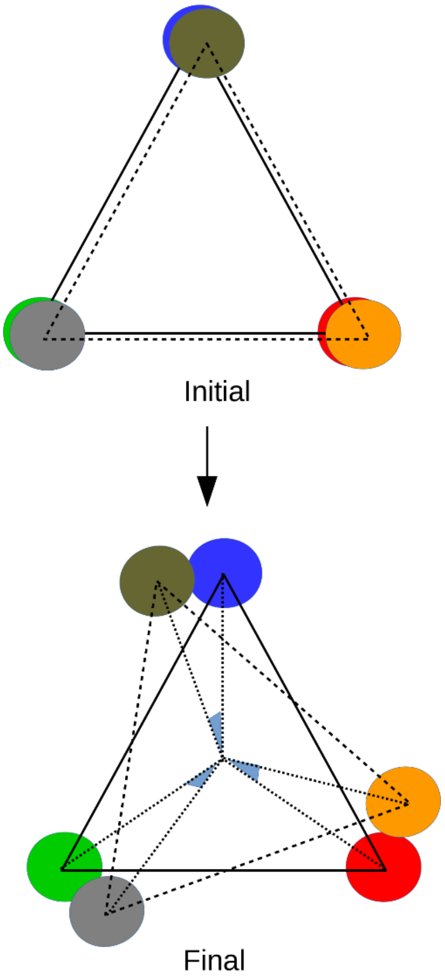

Figure S6. Calculation of rotation angle. The C-terminal domains of each ArgR subunit (colored circles) are located at the apexes of two triangles (solid and dashed lines). Colored circles represent centers of mass of these C-domains (residues 85-149) of the three monomers in each layer of the ArgR hexamer; the layers are shown as slightly offset for clarity but the two trimeric layers are directly stacked. The angle between two triangles in the apoBsArgRC crystal structure is initially zero as shown in the upper panel, as the two trimers stack directly upon each other. To calculate the rotation angle in each frame of the trajectory a triangle representing the centers of monomer mass in that frame was superimposed on the starting structure. Using g_traj and an in-house script (14), the angle of each monomer's center of mass with respect to its corresponding initial position was calculated (blue shaded areas at center) and the average of the resulting deviation of the angles was assigned as the value of rotation.
